# Characterization of urinary cotinine concentrations among non-smoking adults in smoking and smoke-free homes in the Korean national environmental health survey (KoNEHS) cycle 3 (2015–2017)

**DOI:** 10.1186/s12889-021-11265-y

**Published:** 2021-07-06

**Authors:** Jeonghoon Kim, In-Keun Shim, Soo Ran Won, Jungmin Ryu, Jongchun Lee, Hyen-Mi Chung

**Affiliations:** Department, Indoor Environment and Noise Research Division, Environmental Infrastructure Research National Institute of Environmental Research, Seo-gu, Incheon, 22689 Republic of Korea

**Keywords:** Cotinine, KoNEHS, Secondhand smoke, Smoke-free home, Smoking home

## Abstract

**Background:**

Although many indoor public places have implemented smoke-free regulations, private homes have remained sources of tobacco smoke pollutants. This study examined differences in urinary cotinine concentrations in the Korean non-smoking adult population between living in smoking and smoke-free homes, and the relationship of urinary cotinine concentrations with socio-demographic factors in smoke-free homes.

**Methods:**

Samples from 2575 non-smoking adults (≥19 years old) in the Korean National Environmental Health Survey cycle 3 (2015–2017), a representative Korean study, were used. Smoking and smoke-free homes were defined based on whether there were smokers at homes. Weighted linear regression models were used to determine urinary cotinine concentrations and identify factors associated with urinary cotinine.

**Results:**

The geometric mean of urinary cotinine concentrations for non-smoking adults living in smoking homes was 2.1 μg/L (95% confidence interval [CI] = 1.8–2.4), which was significantly higher than the mean of 1.3 μg/L (95% CI = 1.2–1.4) for those living in smoke-free homes. Urinary cotinine concentrations were different significantly by home smoking status in most socio-demographic subgroups. Data from smoke-free home showed urinary cotinine concentration in adults was significantly higher in those who lived in homes with ventilation duration < 30 min/day, those who spent more time indoors at home, those who spent less time outdoors, and those who worked in non-manual or manual occupations.

**Conclusions:**

The urinary cotinine concentration in Korean non-smoking adults living in smoking homes was higher than that in adults living in smoke-free homes. Even in smoke-free homes, home-related factors, such as ventilation duration and time spent indoors, were associated with urinary cotinine concentration. Further study is warranted to examine potential sources of tobacco smoke pollution in smoke-free homes.

## Background

Secondhand smoke (SHS) exposure is causally associated with coronary heart disease, stroke, nasal irritation, and lung cancer in adults, and low birth weight [1]. SHS exposure can cause sudden infant death syndrome, middle-ear disease, respiratory infections, and lower respiratory illness in children [2]. In 2006, there were 42,000 SHS-attributable deaths in America, comprising more than 41,000 adult and 900 infant deaths [3]. Globally, 1% of deaths and 0.7% of disease burden in disability-adjusted life year was attributable to SHS exposure in 2004 [4]. In 2006, the US Surgeon General concluded that there is no risk-free level of SHS exposure [2].

To reduce SHS exposure, many countries have implemented smoke-free regulations in indoor public places or workplaces. In Korea, most indoor public places prohibited smoking in all indoor areas from December 8, 2012. For hospitality venues, smoke-free regulations were gradually enforced based on venue size from 2013 to 2014. Since January 1, 2015 [5] hospitality venues of all sizes implemented smoke-free regulations.

Private indoor places, such as home environments, have limited regulations. When urinary cotinine concentration is used as a biomarker for SHS exposure, it was found to be higher in non-smokers who lived at home with smokers than in those who did not [6, 7]. Even in smoke-free homes, non-smoking residents could be exposed to SHS due to SHS incursion from neighboring units or outside homes [8, 9]. Furthermore, non-smoking residents could be exposed to residual tobacco smoke pollutants, referred to as third-hand smoke (THS) [10], by living in homes previously occupied by smokers [11] or following SHS incursion [12]. However, tobacco smoke pollutant exposure in smoke-free homes has not been well characterized. One study reported that urinary cotinine were detected in 88% of non-smoking Korean adult residents who spent the majority of time at home and did not reside with smokers [13].

In Korea, the National Institute of Environmental Research (NIER) within the Ministry of Environment conduct the Korean National Environmental Health Survey (KoNEHS). The KoNEHS is a national biomonitoring program comprising a cross-sectional study of a representative sample of the population of Korea conducted every 3 years. KoNEHS cycle 3 was conducted between 2015 and 2017. A previous study based on KoNEHS cycle 3 found that the urinary cotinine concentrations were 1.6 times higher in non-smoking adults who resided with smokers in the family home than in those who did not [14].

In this study, we stratified the non-smoking adult population in those who resided with smokers (i.e., living in a smoking home) and those without smokers (i.e., living in a smoke-free home) in KoNEHS cycle 3 to examine differences in urinary cotinine concentration in these populations. The purposes of this study were to determine differences of urinary cotinine concentrations in the Korean non-smoking adult population by those who lived in smoking and smoke-free homes and the relationship of this concentration with socio-demographic factors in smoking and smoke-free homes.

## Methods

### Study population

This study used data from KoNEHS cycle 3 (2015–2017), a representative cross-sectional sample of the population of Korea. KoNEHS cycle 3 was conducted between August 2015 to June 2017 to ensure homogeneity of the sample composition for each year considering regional and seasonal distribution. The study population of KoNEHS cycle 3 comprised 6167 individuals, including 2380 children (≥3 years old) and 3787 adults (≥19 years old). Different multi-stage stratified cluster sampling methods were used between children and adults. In the present study, data from adults were used. For adults, the first stratification was centered on local administrative districts and coastal areas based on the Population and Household Census 2015 provided by Statistics Korea. The second stratification was based on the proportion of residential-complex districts, as well as location within 5 km of the east, south, and west coast, which related to socio-economic status. Ultimately, KoNEHS cycle 3 included 233 districts nationwide, including 20 areas that had national air quality monitoring stations.

The KoNEHS cycle 3 collected questionnaires and urine and blood samples for 16 clinical tests and performed analyses for 26 harmful environmental substances (e.g., phthalates and VOC metabolites). In the present study, the urinary cotinine concentration, the primary metabolite of nicotine, was used as a biomarker for SHS exposure. Cotinine is a specific and sensitive biomarker for SHS exposure with an average half-life of 17 h [15]. This study was approved by the institutional review board of the NIER in Korea (NIER-2016-BR-003-01, NIER-2016-BR-003-03).

Based on questionnaires, the sample of 3787 adult participants was limited to never and former smokers (*n* = 3183). Next, our sample was limited to those who lived in apartments, attached housing, or detached housing (*n* = 3168) because participants who answered “others” could not be distinguished. We then limited our samples to participants whose proportion of daily time spent indoors at home, at the workplace, indoors somewhere other than home or workplace, on transportation, and outdoors as recorded by the questionnaires was more than 80% (i.e., 1152 of 1440 min) (*n* = 3094). Finally, our samples were limited to participants whose urinary creatinine concentrations were between 0.3 and 3.0 g/L [16] (*n* = 2701). Among the 2701 qualified participants, 126 were excluded because their urinary cotinine concentration was higher than the cut-off point of 53 μg/L [17], and they were suspected of being smokers. The cut-off point of 53 μg/L for distinguishing true smokers from true nonsmokers was previously determined in KoNEHS cycle 1 (2009–2011) using the receiver operating characteristic curve, which was 97.1% for sensitivity and 95.1% for specificity [17]. Ultimately, a total of 2575 non-smoking adults were included in the final analysis.

### Smoking home status

Non-smokers were classified as participants who answered “I have never smoked” and “I used to smoke in the past but not anymore.” Smokers were defined as participants who answered “I smoke now” and were excluded from this study. Among the non-smokers, those who lived in smoking homes were defined as those who responded “yes” and those lived in smoke-free homes were defined as those who responded “no” to the question “Do you live with any smokers at home?”

### Socio- demographic characteristics

Socio-demographic characteristics such as sex (male or female), age (19–39, 40–59, or ≥ 60 years), type of housing (apartment, attached housing, or detached housing), household income (< 1000, 1000–1999, 2000–2999, or ≥ 3000 USD/month), ventilation duration at home (< 30, 30–59, 60–599, ≥600 min/day), self-reported weekly SHS exposure (no or yes), time spent in residential indoors (< 780, 780–1079, ≥1080 min/day) and outdoors (< 10, 10–69, ≥70 min/day), and job classification (unemployed, manual occupation, non-manual occupation, or hospitality venue worker). In Korea, an apartment was defined as a high-rise multifamily building more than or equal to five stories and an attached house was a multi-family house less than or equal to four stories. A detached house included single-family and multifamily houses less than or equal to three stories. These housing type is applied in the KoNEHS because it was based on legal classification in Korea.

Levels of household income and ventilation duration at home was divided into quartiles and time spent at residential indoors and outdoors was divided into tertiles based on the non-smoking adult population (*n* = 2575). In the ventilation duration at homes, the ventilation methods included natural ventilation (opening a window and/or front door) and mechanical ventilation (running ventilation fan, or HVAC system). Among the non-smoking respondents who lived at home with ventilation duration more than 0 min/day, most of them used natural ventilation (94%) in their homes. For self-reported weekly SHS exposure, the question was “How often do you smell the cigarette smokes by someone in indoor or enclosed places? ” and respondents chose following option: “no,” “1–2 times/week,” “3–4 times/week,” “5–6 times/week” or “every day.” Due to low percentages of SHS exposure in each category, the question was classified into two categories (i.e., having weekly SHS exposure or not).

For this study, jobs were classified into unemployed (unemployed, students, or stay-at-home parents), non-manual occupations (manager, office/service/sales workers, or expert/related workers), manual occupations (skilled/functional workers, machine operators, assembly/simple labor workers, or agricultural/forestry/fishery workers), and hospitality venues (restaurant, bar, cafe, fast-food franchise, or bakery workers) based on the job-classification code or written job title in the raw data from KoNEHS cycle 3. Similar classifications have been used in a previous study [18]. Jobs in hospitality venues in this study were classified separately from non-manual occupation to examine differences in urinary cotinine concentrations by job type after the implementation of smoke-free regulations in these places started from 1st January, 2015 [5].

### Urinary cotinine

Spot urine samples were collected at survey centers situated throughout the country and frozen at − 20 °C until laboratory analysis following the standard procedure by the NIER [19]. For urinary cotinine analysis, we added 250 μL of internal standard, 50 μL of 0.1 M sodium hydroxide, and 0.5 mL of chloroform to 3-mL urine samples. The solution was centrifuged, and the upper layer was removed. Next, 0.2 g of sodium sulfate was added to remove residual water and 3 μL of solution was injected into a gas chromatograph/mass spectrometer (Clarus 600 T, PerkinElmer, USA) to estimate urinary cotinine concentrations. The detail analytical methods for urinary cotinine have been described in elsewhere [14, 20]. The method detection limit (MDL) for urinary cotinine was 0.3 μg/L. Urinary cotinine concentrations below the MDL were assigned a value of 0.2 μg/L (MDL/ $\sqrt{2}$).

### Statistical analysis

Statistical analyses were conducted using SAS 9.4 (SAS Institute, Inc., Cary, NC, USA). In the analysis, the domain option in SAS was used to control for subgroups with full clusters in reducing the dataset of interest. The PROC SURVEYFREQ function was used to calculate weighted percentages of socio-demographic variables and determine the differences in these percentages between those living in smoking and smoke-free homes.

Natural log (ln)-transformed urinary cotinine and creatinine concentrations were used in statistical analyses due to the skewness of the distribution of the untransformed data. SAS PROC SURVEYMEAN was used to calculate the weighted overall geometric means (GMs) and 95% confidence intervals (CIs) of the urinary cotinine concentrations. The same procedure was used to determine the weighted GM and 95% CI of the urinary cotinine concentrations of non-smoking adults living in smoking and smoke-free homes. Using SAS PROC SURVEYREG, covariate-adjusted least-square geometric means (LSGMs) and 95% CIs of urinary cotinine concentrations of non-smoking adults by subgroup living in smoking and smoke-free homes were estimated in weighted multivariable linear regression models. Socio-demographic variables, including sex, age, type of housing, household income, ventilation duration at home, weekly SHS exposure, time spent at residential indoors and outdoors, and job classification, were included as covariates. Furthermore, ln-transformed urinary creatinine concentrations included in the weighted linear regression models as an independent variable to adjust for hydration. A previous study reported that urinary creatinine concentrations is not only factor that affects hydration but also factor that affects age and several other factors [21]. Model based normalization to adjust for hydration and other factor have been used previously [22]. We also included interactions of the home smoking status with each socio-demographic variables to estimate weighted LSGMs and 95% CIs and to test the differences in urinary cotinine concentrations of non-smoking adults between living in smoking homes and smoke-free homes. The difference test of urinary cotinine concentrations were examined by including the home smoking status variable as a continuous variable in the weighted linear regression models. The false discovery rate (FDR) was controlled at the levels of 0.05 with the Benjamini–Hochberg’s correction for multiple comparison. Among the socio-demographic characteristics, we did not use education level, former smoker status, or time spent at the workplace because of potential collinearity between age and education level (Spearman’s rho = − 0.62), sex and former smoker status (Spearman’s rho = − 0.69), and time spent at residential indoors and that at the workplace (Spearman’s rho = − 0.72). Similar criteria have been used in previous studies [18]. The same SAS procedure was used to conduct weighted multivariable linear regression analysis to examine relationships between urinary cotinine concentrations and socio-demographic characteristics in non-smoking adults who lived in smoking and smoke-free homes. A *p*-value of less than 0.05 was considered statistically significant.

## Results

### Participant characteristics

The characteristics of the non-smoking adults who lived in smoking and smoke-free homes are shown in Table 1. Female non-smoking adults were more likely to live in smoking homes (*p* < 0.001). Non-smoking adults who were younger (*p* < 0.001), those who had higher household income (*p* = 0.004), and those who were exposed to SHS weekly (*p* = 0.002) were more likely to live in smoking homes. Non-smoking adults who were unemployed were more likely to live in smoking homes (*p* < 0.001). However, the type of housing, ventilation duration at home, time spent at residential indoors, and time spent outdoors were not associated with living in a smoking or smoke-free home.

**Table 1:** Proportion of non-smoking adult population by home smoking status^a^

	Total (%)	Smoking home	Smoke-free home	*p*-value^b^
		%	%	
Sex				
Male	948 (41.8)	22.6	49.1	< 0.001
Female	1627 (58.2)	77.4	50.9	
Age (years)				
19–39	541 (34.7)	45.9	30.5	< 0.001
40–59	951 (38.1)	37.0	38.5	
≥ 60	1083 (27.2)	17.1	31.0	
Type of housing				
Apartment	1187 (55.2)	51.9	56.4	0.287
Attached housing	423 (18.2)	21.4	17.0	
Detached housing	965 (26.7)	26.7	26.6	
Household income (USD/month)				
< 1000	510 (12.5)	6.7	14.7	0.004
1000–1999	506 (16.3)	15.9	16.5	
2000–2999	508 (21.7)	21.1	22.0	
≥ 3000	1051 (49.4)	56.3	46.8	
Ventilation duration at home (min/day)				
< 30	518 (19.3)	17.7	19.9	0.485
30–59	449 (16.7)	17.2	16.5	
60–599	953 (38.1)	35.7	38.9	
≥ 600	655 (26.0)	29.5	24.7	
Weekly secondhand smoke exposure				
No	2290 (86.0)	80.6	88.1	0.002
Yes	285 (14.0)	19.4	11.9	
Time spent indoors at home (min/day)				
< 780	596 (27.5)	24.3	28.8	0.164
780–1079	984 (39.1)	38.6	39.3	
≥ 1080	995 (30.9)	37.2	31.9	
Time spent outdoors (min/day)				
< 10	810 (41.9)	34.2	33.7	0.190
10–69	900 (30.6)	36.1	36.0	
≥ 70	865 (27.5)	29.7	30.3	
Job classification				
Unemployed	1144 (42.9)	48.7	40.7	< 0.001
Non-manual occupation	663 (32.7)	32.8	32.7	
Manual occupation	678 (21.1)	14.1	23.8	
Hospitality venue employee^c^	90 (3.3)	4.5	2.8	

^a^ Proportion of variables weighted

^b^ Chi-square test based on the weighted frequency between non-smoking adults living in smoking homes and smoke-free homes

^c^ Participants who worked in restaurants, bars, cafes, fast-food franchises, or bakeries

### Urinary cotinine levels by smoking home status

**Fig. 1:**
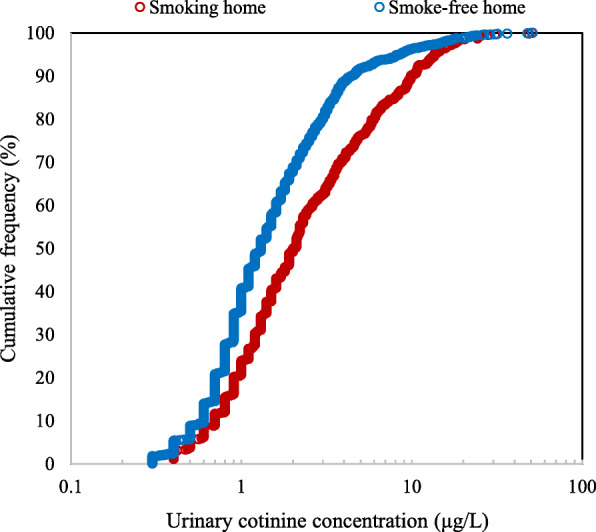
Cumulative frequency of urinary cotinine concentrations in non-smoking adults living in smoking and smoke-free homes. This graph included only the detected urinary sample (*n* = 621) from 641 samples in smoking home and urinary sample (*n* = 1810) from 1934 samples in smoke-free homes

Urinary cotinine was detected in 2422 of 2575 samples (94.1%). The overall GM of the urinary cotinine concentrations of 2575 non-smoking adults was 1.5 μg/L (95% CI = 1.4–1.6). Urinary cotinine was detected in 95.5% of non-smokers living in smoking homes and 93.6% of non-smokers living in smoke-free homes. The distribution of urinary cotinine concentrations among non-smoking adults living in smoking and smoke-free home are shown in Fig. 1. The GM of urinary cotinine concentration for non-smoking adults living in smoking homes was 2.1 μg/L (95% CI = 1.8–2.4) and that for those in smoke-free homes was 1.3 μg/L (95% CI = 1.2–1.4), which was significantly different (*p* < 0.001).

### LSGMs and differences in urinary cotinine levels by smoking home status

The LSGM of urinary cotinine of non-smoking adults by living in smoking and smoke-free homes are shown in Table 2. Cotinine concentrations in the non-smoking-adult population were different significantly between living in smoking homes and smoke-free homes (*p* < 0.001). The concentrations in subgroups, including those based on sex, age, type of housing, household income, ventilation duration at home, weekly SHS exposure, time spent at residential indoor, and time spent at outdoor were different significantly by home smoking status. Regarding job classification, all levels except the hospitality venue employees, showed significant differences by home smoking status.

**Table 2:** Least-square geometric means and differences of urinary cotinine concentrations (μg/L) among non-smoking adults by living in smoking homes and smoke-free homes^a^

	Smoking home	Smoke–free home	*p*-value^c^	FDR *p*-value^d^
	LSGM (95% CI)^b^	LSGM (95% CI)		
Overall	2.2 (1.8–2.7)	1.3 (1.1–1.6)	< 0.001	–
Sex				
Male	2.0 (1.6–2.6)	1.4 (1.1–1.7)	0.001	0.002
Female	2.2 (1.8–2.7)	1.3 (1.1–1.5)	< 0.001	0.003
Age (years)				
19–39	2.1 (1.6–2.7)	1.2 (1.0–1.5)	< 0.001	0.001
40–59	2.4 (1.9–2.9)	1.4 (1.2–1.7)	< 0.001	< 0.001
≥ 60	2.0 (1.6–2.6)	1.4 (1.2–1.7)	0.003	0.004
Type of housing				
Apartment	2.3 (1.9–2.8)	1.3 (1.1–1.5)	< 0.001	< 0.001
Attached housing	1.7 (1.2–2.5)	1.4 (1.1–1.8)	0.227	0.236
Detached housing	2.4 (1.9–3.2)	1.5 (1.2–1.8)	< 0.001	< 0.001
Household income (USD/month)				
< 1000	2.1 (1.4–3.0)	1.3 (1.0–1.5)	0.006	0.008
1000–1999	2.3 (1.7–3.3)	1.5 (1.2–1.9)	0.012	0.014
2000–2999	2.4 (1.7–3.5)	1.3 (1.1–1.6)	0.002	0.002
≥ 3000	2.0 (1.6–2.6)	1.3 (1.1–1.6)	< 0.001	< 0.001
Ventilation duration at home (min/day)				
< 30	2.0 (1.5–2.7)	1.5 (1.3–1.9)	0.038	0.040
30–59	2.4 (1.6–3.5)	1.4 (1.1–1.6)	0.005	0.006
60–599	2.0 (1.6–2.5)	1.2 (1.0-1.5)	< 0.001	< 0.001
≥ 600	2.3 (1.7–3.1)	1.3 (1.0-1.7)	< 0.001	< 0.001
Weekly secondhand smoke exposure				
No	2.1 (1.7–2.6)	1.3 (1.1–1.5)	< 0.001	< 0.001
Yes	2.3 (1.7–3.3)	1.4 (1.1–1.7)	0.001	0.002
Time spent at residential indoor (min/day)				
< 780	2.3 (1.8–3.0)	1.2 (1.0–1.5)	< 0.001	< 0.001
780–1079	2.0 (1.6–2.5)	1.3 (1.1–1.6)	< 0.001	< 0.001
≥ 1080	2.4 (1.8–3.2)	1.5 (1.2–1.8)	< 0.001	< 0.001
Time spent at outdoor (min/day)				
< 10	2.4 (1.9–3.1)	1.4 (1.2–1.8)	< 0.001	< 0.001
10–69	2.1 (1.7–2.7)	1.4 (1.2–1.7)	< 0.001	< 0.001
≥ 70	2.2 (1.6–2.9)	1.2 (1.0–1.4)	< 0.001	< 0.001
Job classification				
Unemployed	2.5 (2.0–3.2)	1.3 (1.1–1.5)	< 0.001	< 0.001
Non–manual occupation	1.9 (1.5–2.3)	1.4 (1.2–1.7)	0.019	0.022
Manual occupation	2.1 (1.6–2.7)	1.4 (1.2–1.6)	< 0.001	0.001
Hospitality venues employee^e^	1.8 (1.1–3.1)	1.5 (0.7–3.2)	0.685	0.685

^a^ Weighted multivariable linear regression analyses were used to estimate least-square geometric means (LSGMs) and 95% confidence intervals (CIs) of urinary cotinine concentration

^b^ LSGMs of urinary cotinine concentrations were adjusted for all variables listed in the table and ln-transformed creatinine concentrations

^c^ An interaction term between home smoking status (continuous) and each socio-demographic variable (categorical) (e.g., sex × home smoking status) in the linear regression models was added to examine differences in urinary cotinine concentrations between smoking homes and smoke-free homes while controlling for socio-demographic factors and ln-transformed creatinine concentrations

^d^ False discovery rate (FDR) corrected *p*-values for multiple testing.

^e^ Participants who worked in restaurants, bars, cafes, fast-food franchises, or bakeries where smoke-free regulations have been implemented since 2015

### Factors associated with urinary cotinine levels by smoking home status

Among non-smoking adults living in smoking homes, urinary cotinine concentrations were significantly lower in non-smoking adults who worked in non-manual occupations than those who were unemployed (*p* = 0.002, Table 3). However, sex, age, type of housing, household income, ventilation duration at home, weekly SHS exposure, time spent at residential indoors, and time spent outdoors were not associated with urinary cotinine concentrations.

**Table 3:** Factors associated with urinary cotinine concentrations (μg/L) among non-smoking adults living in smoking homes and smoke-free homes^a^

	Smoking home (*n* = 641)			Smoke-free home (*n* = 1934)		
	*β*	SE	*p*-value	*β*	SE	*p*-value
Sex						
Male	− 0.14	0.13	0.279	0.05	0.07	0.498
Female	Reference			Reference		
Age (years)						
19–39	−0.17	0.13	0.193	− 0.11	0.07	0.113
40–59	Reference			Reference		
≥ 60	−0.24	0.14	0.093	0.07	0.07	0.341
Type of housing						
Apartment	Reference			Reference		
Attached housing	−0.23	0.20	0.256	0.09	0.13	0.457
Detached housing	0.08	0.14	0.561	0.12	0.09	0.187
Household income (USD/month)						
< 1000	−0.03	0.26	0.908	−0.08	0.10	0.396
1000–1999	0.03	0.22	0.904	0.09	0.11	0.439
2000–2999	Reference			Reference		
≥ 3000	−0.18	0.17	0.297	−0.02	0.09	0.847
Ventilation duration at home (min/day)						
< 30	−0.01	0.16	0.949	0.20	0.08	0.016
30–59	0.15	0.19	0.434	0.08	0.08	0.274
60–599	Reference			Reference		
≥ 600	0.13	0.17	0.426	0.05	0.10	0.657
Weekly secondhand smoke exposure						
No	Reference			Reference		
Yes	0.14	0.16	0.375	0.07	0.09	0.446
Time spent at residential indoor (min/day)						
< 780	0.19	0.14	0.174	−0.13	0.08	0.101
780–1079	Reference			Reference		
≥ 1080	−0.08	0.14	0.599	0.20	0.07	0.003
Time spent at outdoor (min/day)						
< 10	0.12	0.13	0.336	−0.02	0.08	0.792
10–69	Reference			Reference		
≥ 70	−0.11	0.14	0.447	−0.19	0.07	0.005
Job classification						
Unemployed	Reference			Reference		
Non–manual occupation	−0.49	0.16	0.002	0.20	0.08	0.011
Manual occupation	−0.32	0.17	0.066	0.16	0.08	0.041
Hospitality venue employee^b^	−0.60	0.35	0.087	0.25	0.36	0.490

^a^ R^2^ values from multivariable linear regression models were 0.12 for smoking homes and 0.16 for smoke-free homes after adjusting for variables listed in the table and ln-transformed creatinine concentrations

^b^ Participants who worked in restaurants, bars, cafes, fast-food franchises, or bakeries where smoke-free regulations have been implemented since 2015

For smoke-free homes, urinary cotinine concentrations were significantly higher in non-smoking adults who lived at home with ventilation duration < 30 min/day than those with ventilation duration 60–599 min/day (*p* = 0.016). Urinary cotinine concentrations were significantly higher in non-smoking adults who spent ≥1080 min/day indoors at home than those who spent 780–1079 min/day indoors at home (*p* = 0.003). Urinary cotinine concentrations were significantly lower in non-smoking adults who spent ≥70 min/day outdoors than those who spent 10–69 min/day outdoors (*p* = 0.005). Urinary cotinine concentrations were significantly higher in non-smoking adults who worked in non-manual (*p* = 0.011) and manual occupations than those who were unemployed (*p* = 0.041). However, sex, age, type of housing, household income, and weekly SHS exposure were not significantly associated with urinary cotinine.

## Discussion

This study examined factors associated with urinary cotinine concentrations in non-smoking adults living in smoking and smoke-free homes. We further examined the effects of the implementation of smoke-free regulations after the regulation of smoking in all hospitality venues using differences in urinary cotinine concentrations of non-smoking adults who worked in these venues. Because this study analyzed a weighted sample, the results are representative of the non-smoking adult population in Korea. Urinary cotinine concentrations were used as an objective measure of SHS exposure.

GM of urinary cotinine concentrations that measured as free cotinine in Korean non-smoking adult populations (1.5 μg/L, 95% CI = 1.4–1.6, *n* = 2575) in this study was slightly different from that in previously published studies in other countries. Based on the U.S. National Health and Nutrition Examination Survey 2013–2014, GM of total urinary cotinine concentrations, including free and conjugated cotinine, of non-smoking adult (≥20 years) were 0.87 μg/L (95% CI = 0.54–1.42) [22]. A national representative study from Canada conducted between 2012 and 2013 showed that urinary cotinine concentrations that measured as free cotinine in most non-smokers aged 12–79 years (89%) were below a detection limit of 1.1 μg/L [23]. No comparable recent nationally representative data of urinary cotinine concentrations in non-smokers in other countries have been reported.

Urinary cotinine concentrations in adults who lived in smoking and smoke-free home were significantly different. Overall, the GMs and LSGMs of the urinary cotinine concentrations were 1.6 and 1.7 times, respectively, higher in non-smoking adults who lived in smoking homes than those who lived in smoke-free homes. Similarly, the urinary cotinine concentrations of most subgroups, including those based on sex, age, type of housing, household income, ventilation duration at home, weekly SHS exposure, time spent at residential indoor, time spent at outdoor, and job classification were significantly different by home smoking status. This finding indicates that homes with smokers are a significant source of tobacco smoke pollutants for non-smoking residents. Similar findings have been reported in previous studies. Among Korean non-smoking adult women who spent more than 19 h a day at home, urinary cotinine concentrations were higher in those who lived with smokers at home (9.96 μg/g creatinine, *n* = 26) than those who lived without smokers at home (7.53 μg/g creatinine, *n* = 31, *p* = 0.133) in 1999 [24]. A study in eastern Germany conducted in 1998–1999 showed that urinary cotinine concentrations were higher in school children who lived with one (GM = 6.9 μg/L, 95% CI = 6.0–7.9) or more than one smoker at home (GM = 10.4 μg/L, 95% CI = 8.8–12.2) than those in who did not (GM = 2.7 μg/L, 2.5–2.8) [25].

In the multivariable analysis, the urinary cotinine concentration in those living in smoking homes was only associated with job classification. Urinary cotinine concentrations were 24% lower in non-smoking adults who worked in non-manual occupations than in those who were unemployed. This might be because non-smoking adults who were unemployed spent more time at home than those who worked in any other type of job; therefore, they might be more exposed to SHS in their home. That other factors except for job classification were not associated with urinary cotinine concentration indicates that homes with smokers are significant contributors to the urinary cotinine concentration in non-smokers.

Unlike smoking homes, urinary cotinine concentrations were associated with several factors in smoke-free homes. Urinary cotinine concentrations in smoke-free homes were associated with home-related factors, such as ventilation duration, time spent at indoors at home, and time spent outdoors. Urinary cotinine concentrations were higher in non-smoking adults who lived in less ventilated homes, those who spent a long time indoors at home, and those who spent less time outdoors. This might be that they were exposed to SHS due to incursion from neighboring units or outside homes [9] or THS in their homes [10]. These tobacco smoke pollutants might accumulate inside homes due to the reduced ventilation duration, which might contribute to urinary cotinine levels in non-smoking adults. Furthermore, non-smoking adults who spent more time at home might be more exposed to tobacco smoke pollutants in their homes. Frequent ventilation at home could reduce tobacco smoke pollution exposure in non-smokers in their homes.

Job classification was associated with urinary cotinine concentration in people who lived in smoke-free homes. Urinary cotinine concentrations were 1.1 times higher in non-smoking adults who worked in both non-manual and manual occupations than those who were unemployed. Although many indoor public places have implemented smoke-free regulations, some non-manual occupational spaces, such as small office buildings < 1000 m^2^, or manual occupational spaces were not included in smoke-free regulations [26]. These findings indicate that comprehensive smoke-free regulations for indoor places are needed to further reduce SHS exposure to non-smokers.

It is interesting to note that urinary cotinine concentrations in non-smoking adults who worked in hospitality venues were not significantly higher than in those with other job types in for both adults who lived in smoking homes and those who lived in smoke-free homes. This might be because smoke-free regulations have been implemented in all hospitality venues since 2015. In Korea, partial smoke-free regulations were implemented in hospitality venues based on their size; the regulations were implemented in hospitality venues ≥150 m^2^ in July 2013, in venues ≥100 m^2^ in January 2014, and in all venues in January 2015 [5]. A previous study that used data from 2014 in KoNEHS cycle 2 (2010–2014), when smoke-free regulations were implemented in hospitality venues ≥100 m^2^, showed that urinary cotinine concentrations were significantly higher in the Korean non-smoking adult population who worked at hospitality venues (LSGM = 2.32 μg/g creatinine, 95% CI = 1.45–3.71) than in those who were unemployed (LSGM = 1.24 μg/g creatinine, 95% CI = 1.01–1.53) [18]. These findings suggest that the implementation of smoke-free regulations in all hospitality venues without exception was effective in reducing SHS exposure in non-smoking workers in hospitality venues.

This study used cotinine in the urine as a biomarker for SHS exposure. Cotinine concentrations can also be measured in other specimens, such as in the saliva or blood (e.g., serum or plasma) [15]. The cotinine concentration in the urine is more sensitive in measuring low levels of SHS exposure than that in the saliva or blood because the cotinine concentration in the urine are four- to six-fold higher than that in the saliva or blood [27, 28]. In addition, urine sample collection is less invasive than saliva or blood collection. However, urinary cotinine concentrations require creatinine based adjustment for renal clearance of cotinine because urinary cotinine concentrations can be affected by several factors, including urine pH, renal function, and urine flow rate. On the other hand, hydration adjustments for serum and plasma cotinine are not required [27]. In the present study, linear regression model based normalization by adding ln-transformed urinary creatininie as an indepentant variable were used to adjust for hydration, similar to previosu studies [22].

This study has several limitations. Some non-smoking adults that were classified as having non-manual occupations might have worked in hospitality venues that smoke-free regulations did not cover (e.g., karaoke venues that serve alcohol or nightclubs). However, the effects of these occupations on urinary cotinine levels were minimal because few subjects worked in such places. This study could not consider potential sources of nicotine, including nicotine replacement therapy or smokeless tobacco. Although non-smokers were selected based on questionnaires and cut-off levels of urinary cotinine, they might include those who use electronic nicotine delivery devices. Home smoking status in the present study were determined by non-smoking adult respondents who answered they resided with or without smokers in their homes. The non-smoking respondents in smoke-free homes, defined by questions, might resided with occasional smokers in their homes. For smoking homes, we could not identify whether these smokers smoked inside the home, quantify the number of cigarettes smoked by smokers, or number of smokers in their homes. The present study did not take into account potential SHS from the workpalces in our analysis because the questionnaires in KoNEHS cycle 3 did not ask frequency of SHS in specific workplaces. Home smoking bans, building smoking bans, and implementation or violation of smoking bans in buildings could be important variable for urinary cotinine levels in non-smoking adults. Unfortunately, the KoNEHS cycle 3 did not measure these variables. These unmeasured factors might over-or underestimate urinary cotinine levels in non-smoking adults.

## Conclusions

Overall, 2575 non-smoking adults in KoNEHS cycle 3 (2015–2017) were included in the analysis. Urinary cotinine concentrations were significantly higher in non-smoking adults living in smoking homes than those living in smoke-free homes. This indicates that homes with smokers are a significant source of tobacco smoke pollutant exposure. In the multivariable analysis, only job classification was associated with urinary cotinine concentrations in those living in smoking homes. For smoke-free homes, home-related factors, including ventilation duration at home, time spent indoors at home, and time spent outdoors, were associated with urinary cotinine concentration. These findings suggest that there are potential sources of tobacco smoke pollutants even in smoke-free homes. Further studies are needed to examine sources of tobacco smoke pollution at home.

## Data Availability

KoNEHS cycle 3 (2015–2017) data can be obtained after the judgment of the raw data request form in National Institutes of Research (NIER) in Korea. Obtained raw data cannot be shared with the public. The data can be requested via NIER by calling + 82–032–560-7129.

## Abbreviations

SHS: Secondhand smoke
THS: Third-hand smoke
NIER: National Institute of Environmental Research
KoNEHS: Korean National Environmental Health Survey
MDL: Method detection limit
GM: Geometric mean
CI: Confidence interval
LSGM: Least-square geometric mean
FDR: False discovery rate
